# Supplementary material for: Burden of disease and productivity impact of Streptococcus suis infection in Thailand
Source: PLoS Negl Trop Dis. 2021 Jan 22;15(1):e0008985. doi: 10.1371/journal.pntd.0008985 (PMC7857555; doi:10.1371/journal.pntd.0008985)
Supplement: S1 Table — (DOCX) [file pntd.0008985.s001.docx]

**S1 Table.** A data set of 133 *Streptococcus suis* patients admitted with total IPD cost from Office of medical records and statistics, CMU Hospital

| studyid | ageyears | Admission date | cpi | cpi2019 | adjtotalcost2019 | drugcost | labcost | Xray | other | totalcost |
| --- | --- | --- | --- | --- | --- | --- | --- | --- | --- | --- |
| 1 | 47 | 8/1/2016 | 100.91 | 103.08 | 42509.90189 | 5151 | 9920 | 6150 | 20394 | 41615 |
| 2 | 62 | 11/26/2017 | 101.5 | 103.08 | 806584.2432 | 134027 | 76510 | 59880 | 523804 | 794221 |
| 3 | 32 | 10/12/2017 | 101.5 | 103.08 | 269095.6717 | 49381 | 22220 | 2250 | 191120 | 264971 |
| 4 | 55 | 9/26/2016 | 100.91 | 103.08 | 290522.9765 | 55612 | 19360 | 1980 | 207455 | 284407 |
| 5 | 65 | 9/20/2017 | 101.5 | 103.08 | 101578.9927 | 2692 | 14370 | 21090 | 61870 | 100022 |
| 6 | 89 | 5/24/2017 | 101.5 | 103.08 | 76809.32571 | 7455 | 13575 | 1530 | 53072 | 75632 |
| 7 | 85 | 5/5/2017 | 101.5 | 103.08 | 377313.4227 | 122662 | 8005 | 10900 | 229963 | 371530 |
| 8 | 71 | 5/22/2017 | 101.5 | 103.08 | 302721.0786 | 110033 | 16955 | 1720 | 169373 | 298081 |
| 9 | 46 | 10/2/2016 | 100.91 | 103.08 | 78776.36944 | 5293 | 14900 | 6150 | 50775 | 77118 |
| 10 | 54 | 7/20/2016 | 100.91 | 103.08 | 21544.54742 | 7022 | 1740 | 720 | 11609 | 21091 |
| 11 | 29 | 7/8/2016 | 100.91 | 103.08 | 80531.31384 | 11522 | 12310 | 3030 | 51974 | 78836 |
| 12 | 65 | 6/15/2016 | 100.91 | 103.08 | 155908.1169 | 23092 | 17880 | 31230 | 80424 | 152626 |
| 13 | 49 | 11/14/2016 | 100.91 | 103.08 | 350528.1827 | 91361 | 16900 | 9600 | 225288 | 343149 |
| 14 | 52 | 8/8/2016 | 100.91 | 103.08 | 78213.52056 | 5525 | 16720 | 1310 | 53012 | 76567 |
| 15 | 66 | 7/18/2016 | 100.91 | 103.08 | 535166.1084 | 188351 | 25775 | 1500 | 308274 | 523900 |
| 16 | 51 | 10/5/2015 | 100 | 103.08 | 427842.8172 | 102161 | 20350 | 3420 | 289128 | 415059 |
| 17 | 64 | 7/1/2015 | 100 | 103.08 | 321644.6472 | 93523 | 21510 | 24660 | 172341 | 312034 |
| 18 | 62 | 6/4/2015 | 100 | 103.08 | 37713.8796 | 2810 | 10220 | 4440 | 19117 | 36587 |
| 19 | 56 | 6/3/2015 | 100 | 103.08 | 174729.8772 | 14403 | 9910 | 48630 | 96566 | 169509 |
| 20 | 53 | 5/22/2015 | 100 | 103.08 | 295564.3764 | 95215 | 21895 | 18660 | 150963 | 286733 |
| 21 | 55 | 5/10/2015 | 100 | 103.08 | 50065.956 | 8556 | 13540 | 5040 | 21434 | 48570 |
| 22 | 61 | 5/6/2015 | 100 | 103.08 | 31365.1824 | 12103 | 8025 | 1310 | 8990 | 30428 |
| 23 | 57 | 4/30/2015 | 100 | 103.08 | 73029.0876 | 8437 | 5690 | 2250 | 54470 | 70847 |
| 24 | 65 | 2/13/2015 | 100 | 103.08 | 84313.2552 | 12784 | 14620 | 11280 | 43110 | 81794 |
| 25 | 50 | 5/18/2014 | 98.58 | 103.08 | 18425.36701 | 2241 | 6200 | 270 | 8910 | 17621 |
| 26 | 54 | 3/11/2014 | 98.58 | 103.08 | 56412.72063 | 6660 | 3610 | 10410 | 33270 | 53950 |
| 27 | 26 | 2/6/2014 | 98.58 | 103.08 | 186382.6099 | 13867 | 18550 | 57390 | 88439 | 178246 |
| 28 | 58 | 8/13/2013 | 97.23 | 103.08 | 47023.69022 | 8049 | 3090 | 8660 | 24556 | 44355 |
| 29 | 47 | 8/23/2014 | 98.58 | 103.08 | 51061.09312 | 3395 | 3710 | 9870 | 31857 | 48832 |
| 30 | 90 | 4/9/2014 | 98.58 | 103.08 | 16705.27572 | 2514 | 4905 | 520 | 8037 | 15976 |
| 31 | 55 | 10/25/2012 | 96.29 | 103.08 | 25970.72178 | 5843 | 2255 | 8000 | 8162 | 24260 |
| 32 | 52 | 10/23/2012 | 96.29 | 103.08 | 65664.39007 | 16164 | 9930 | 600 | 34645 | 61339 |
| 33 | 52 | 10/4/2012 | 96.29 | 103.08 | 27741.35549 | 4172 | 7815 | 4900 | 9027 | 25914 |
| 34 | 61 | 10/3/2012 | 96.29 | 103.08 | 47798.54606 | 6600 | 3090 | 9100 | 25860 | 44650 |
| 35 | 46 | 7/24/2012 | 96.29 | 103.08 | 260692.0926 | 93196 | 30325 | 3520 | 116479 | 243520 |
| 36 | 46 | 7/8/2012 | 96.29 | 103.08 | 88665.50005 | 25715 | 8750 | 300 | 48060 | 82825 |
| 37 | 75 | 5/6/2012 | 96.29 | 103.08 | 15233.4448 | 1793 | 5965 | 220 | 6252 | 14230 |
| 38 | 55 | 11/27/2012 | 96.29 | 103.08 | 17292.04736 | 583 | 3910 | 3700 | 7960 | 16153 |
| 39 | 47 | 10/9/2012 | 96.29 | 103.08 | 23566.34251 | 2837 | 9215 | 220 | 9742 | 22014 |
| 40 | 64 | 9/7/2012 | 96.29 | 103.08 | 34327.17084 | 4964 | 9665 | 1340 | 16097 | 32066 |
| 41 | 56 | 7/18/2012 | 96.29 | 103.08 | 19824.88857 | 5190 | 5575 | 220 | 7534 | 18519 |
| 42 | 37 | 6/30/2012 | 96.29 | 103.08 | 41126.0189 | 5285 | 6505 | 13040 | 13587 | 38417 |
| 43 | 51 | 6/25/2012 | 96.29 | 103.08 | 29636.16907 | 3107 | 3135 | 12600 | 8842 | 27684 |
| 44 | 56 | 3/28/2012 | 96.29 | 103.08 | 34001.73393 | 4049 | 6115 | 10020 | 11578 | 31762 |
| 45 | 69 | 11/3/2011 | 95.38 | 103.08 | 264676.1103 | 85467 | 7445 | 4000 | 147993 | 244905 |
| 46 | 51 | 8/2/2011 | 95.38 | 103.08 | 267005.0828 | 21179 | 16795 | 26190 | 182896 | 247060 |
| 47 | 51 | 6/10/2011 | 95.38 | 103.08 | 38760.37115 | 5528 | 6890 | 300 | 23147 | 35865 |
| 48 | 79 | 5/31/2011 | 95.38 | 103.08 | 233566.2248 | 30983 | 25490 | 19300 | 140346 | 216119 |
| 49 | 53 | 4/27/2011 | 95.38 | 103.08 | 8633.949675 | 1787 | 840 | 170 | 5192 | 7989 |
| 50 | 48 | 6/3/2011 | 95.38 | 103.08 | 18187.60034 | 3134 | 3530 | 810 | 9355 | 16829 |
| 51 | 47 | 12/30/2010 | 94.81 | 103.08 | 45601.56566 | 9756 | 13035 | 1090 | 18062 | 41943 |
| 52 | 54 | 9/3/2010 | 94.81 | 103.08 | 29532.34933 | 11101 | 5820 | 520 | 9722 | 27163 |
| 53 | 78 | 5/28/2010 | 94.81 | 103.08 | 34851.06423 | 2009 | 5730 | 1520 | 22796 | 32055 |
| 54 | 53 | 5/20/2010 | 94.81 | 103.08 | 119808.0759 | 29761 | 6345 | 740 | 73350 | 110196 |
| 55 | 55 | 1/18/2010 | 94.81 | 103.08 | 30192.29617 | 3448 | 3605 | 9970 | 10747 | 27770 |
| 56 | 47 | 1/4/2010 | 94.81 | 103.08 | 11016.87206 | 3296 | 3135 | 220 | 3482 | 10133 |
| 57 | 73 | 12/25/2009 | 94.71 | 103.08 | 86986.19829 | 15647 | 8370 | 220 | 55686 | 79923 |
| 58 | 64 | 10/17/2009 | 94.71 | 103.08 | 17582.69876 | 2533 | 5540 | 520 | 7562 | 16155 |
| 59 | 55 | 10/1/2009 | 94.71 | 103.08 | 58078.95724 | 20314 | 5335 | 6900 | 20814 | 53363 |
| 60 | 76 | 8/31/2009 | 94.71 | 103.08 | 10499.55401 | 1325 | 2080 | 560 | 5682 | 9647 |
| 61 | 50 | 9/28/2009 | 94.71 | 103.08 | 265757.2404 | 61561 | 29915 | 9030 | 143672 | 244178 |
| 62 | 65 | 9/8/2009 | 94.71 | 103.08 | 44417.67374 | 21049 | 3220 | 9500 | 7042 | 40811 |
| 63 | 56 | 7/4/2009 | 94.71 | 103.08 | 368854.6544 | 101194 | 29805 | 3580 | 204325 | 338904 |
| 64 | 65 | 6/30/2009 | 94.71 | 103.08 | 25136.02154 | 13228 | 4660 | 520 | 4687 | 23095 |
| 65 | 89 | 6/29/2009 | 94.71 | 103.08 | 66286.39341 | 19138 | 2505 | 740 | 38521 | 60904 |
| 66 | 70 | 6/16/2009 | 94.71 | 103.08 | 253406.3605 | 69466 | 37215 | 3560 | 122589 | 232830 |
| 67 | 48 | 5/29/2009 | 94.71 | 103.08 | 45230.6899 | 10366 | 6700 | 10620 | 13872 | 41558 |
| 68 | 79 | 5/30/2009 | 94.71 | 103.08 | 1063696.136 | 482588 | 68385 | 16700 | 409652 | 977325 |
| 69 | 52 | 5/28/2009 | 94.71 | 103.08 | 33319.51346 | 7273 | 5055 | 5290 | 12996 | 30614 |
| 70 | 64 | 5/23/2009 | 94.71 | 103.08 | 17496.71714 | 1989 | 5405 | 220 | 8462 | 16076 |
| 71 | 56 | 4/16/2009 | 94.71 | 103.08 | 41068.74374 | 4011 | 8000 | 510 | 25213 | 37734 |
| 72 | 56 | 4/10/2009 | 94.71 | 103.08 | 26353.91321 | 3159 | 3725 | 4770 | 12560 | 24214 |
| 73 | 71 | 4/7/2009 | 94.71 | 103.08 | 137111.3107 | 38577 | 2920 | 12170 | 72311 | 125978 |
| 74 | 51 | 4/4/2009 | 94.71 | 103.08 | 47403.08647 | 8798 | 4490 | 8510 | 21756 | 43554 |
| 75 | 74 | 4/3/2009 | 94.71 | 103.08 | 353975.4793 | 18326 | 34145 | 31350 | 241412 | 325233 |
| 76 | 59 | 3/12/2009 | 94.71 | 103.08 | 404862.4542 | 165538 | 11985 | 2140 | 192325 | 371988 |
| 77 | 54 | 3/8/2009 | 94.71 | 103.08 | 21507.37916 | 5356 | 4660 | 510 | 9235 | 19761 |
| 78 | 44 | 4/7/2008 | 94.32 | 103.08 | 11923.26972 | 840 | 2340 | 4600 | 3130 | 10910 |
| 79 | 65 | 9/19/2008 | 94.32 | 103.08 | 11337.48855 | 1287 | 5145 | 170 | 3772 | 10374 |
| 80 | 32 | 9/18/2008 | 94.32 | 103.08 | 41624.34224 | 5599 | 7875 | 5280 | 19333 | 38087 |
| 81 | 42 | 11/23/2008 | 94.32 | 103.08 | 42017.77735 | 3674 | 4575 | 4900 | 25298 | 38447 |
| 82 | 56 | 8/11/2008 | 94.32 | 103.08 | 28222.41221 | 4182 | 7470 | 0 | 14172 | 25824 |
| 83 | 49 | 7/28/2008 | 94.32 | 103.08 | 321334.8511 | 45551 | 52440 | 4030 | 192006 | 294027 |
| 84 | 47 | 7/11/2008 | 94.32 | 103.08 | 35482.38295 | 6171 | 4675 | 170 | 21451 | 32467 |
| 85 | 67 | 12/31/2007 | 93.84 | 103.08 | 18367.44118 | 1529 | 3275 | 5110 | 6807 | 16721 |
| 86 | 44 | 9/9/2007 | 93.84 | 103.08 | 22989.78389 | 3114 | 4395 | 5110 | 8310 | 20929 |
| 87 | 47 | 7/24/2007 | 93.84 | 103.08 | 29929.88875 | 4152 | 3790 | 170 | 19135 | 27247 |
| 88 | 55 | 5/4/2007 | 93.84 | 103.08 | 53544.69949 | 4802 | 6325 | 650 | 36968 | 48745 |
| 89 | 53 | 4/17/2007 | 93.84 | 103.08 | 729126.2302 | 273297 | 56120 | 20130 | 314221 | 663768 |
| 90 | 56 | 4/17/2007 | 93.84 | 103.08 | 96212.39386 | 49222 | 14770 | 2140 | 21456 | 87588 |
| 91 | 42 | 3/18/2007 | 93.84 | 103.08 | 291037.3286 | 66178 | 34045 | 7080 | 157646 | 264949 |
| 92 | 56 | 3/1/2007 | 93.84 | 103.08 | 18237.82225 | 3214 | 3535 | 4770 | 5084 | 16603 |
| 93 | 56 | 1/11/2018 | 102.42 | 103.08 | 193332.8705 | 10270 | 27190 | 21630 | 133005 | 192095 |
| 94 | 60 | 3/1/2018 | 102.42 | 103.08 | 19809.83831 | 1000 | 3380 | 8000 | 7303 | 19683 |
| 95 | 63 | 3/25/2018 | 102.42 | 103.08 | 600020.0548 | 70482 | 71380 | 66750 | 387566.3 | 596178.3 |
| 96 | 48 | 4/25/2018 | 102.42 | 103.08 | 166141.5199 | 17469.25 | 11740 | 35910 | 99958.5 | 165077.8 |
| 97 | 84 | 4/16/2018 | 102.42 | 103.08 | 42099.55477 | 15654 | 8570 | 660 | 16946 | 41830 |
| 98 | 61 | 8/22/2018 | 102.42 | 103.08 | 21191.18278 | 1108 | 5540 | 4080 | 10327.5 | 21055.5 |
| 99 | 41 | 2/15/2006 | 93.45 | 103.08 | 208564.6485 | 28954 | 11170 | 960 | 147996 | 189080 |
| 100 | 41 | 4/2/2006 | 93.45 | 103.08 | 18412.10658 | 5513 | 2480 | 3620 | 5079 | 16692 |
| 101 | 51 | 9/24/2018 | 102.42 | 103.08 | 148310.3506 | 10330.75 | 21900 | 44820 | 70310 | 147360.8 |
| 102 | 80 | 7/26/2006 | 93.45 | 103.08 | 35996.92584 | 3971 | 9370 | 360 | 18933 | 32634 |
| 103 | 27 | 8/8/2006 | 93.45 | 103.08 | 17211.98844 | 2320 | 4580 | 3740 | 4964 | 15604 |
| 104 | 51 | 8/26/2006 | 93.45 | 103.08 | 8517.750241 | 848 | 4360 | 120 | 2394 | 7722 |
| 105 | 25 | 11/24/2006 | 93.45 | 103.08 | 52003.2809 | 5208 | 13960 | 6680 | 21297 | 47145 |
| 106 | 56 | 12/28/2006 | 93.45 | 103.08 | 45200.77303 | 14071 | 8005 | 1120 | 17782 | 40978 |
| 107 | 70 | 5/19/2005 | 92.38 | 103.08 | 16591.21585 | 4584 | 2930 | 120 | 7235 | 14869 |
| 108 | 53 | 5/20/2005 | 92.38 | 103.08 | 13577.36999 | 3698 | 3340 | 240 | 4890 | 12168 |
| 112 | 45 | 6/10/2005 | 92.38 | 103.08 | 28877.57523 | 8220 | 4980 | 3800 | 8880 | 25880 |
| 113 | 66 | 5/27/2005 | 92.38 | 103.08 | 114596.4395 | 6160 | 10450 | 720 | 85371 | 102701 |
| 114 | 54 | 6/9/2005 | 92.38 | 103.08 | 239603.535 | 26444 | 25410 | 19320 | 143558 | 214732 |
| 115 | 71 | 6/10/2005 | 92.38 | 103.08 | 14114.08227 | 1865 | 3400 | 120 | 7264 | 12649 |
| 116 | 52 | 6/14/2005 | 92.38 | 103.08 | 25494.39099 | 5323 | 6700 | 3620 | 7205 | 22848 |
| 117 | 76 | 6/25/2005 | 92.38 | 103.08 | 36185.11929 | 13382 | 4320 | 4220 | 10507 | 32429 |
| 118 | 33 | 6/25/2005 | 92.38 | 103.08 | 4718.827885 | 384 | 3610 | 120 | 115 | 4229 |
| 119 | 81 | 7/12/2005 | 92.38 | 103.08 | 20099.37259 | 4631 | 5990 | 720 | 6672 | 18013 |
| 120 | 67 | 7/15/2005 | 92.38 | 103.08 | 8468.003031 | 1190 | 1790 | 240 | 4369 | 7589 |
| 121 | 78 | 7/16/2005 | 92.38 | 103.08 | 198277.8056 | 64836 | 23820 | 4820 | 84220 | 177696 |
| 122 | 61 | 7/19/2005 | 92.38 | 103.08 | 51903.75926 | 8471 | 6510 | 3620 | 27915 | 46516 |
| 123 | 43 | 8/2/2005 | 92.38 | 103.08 | 19242.41827 | 3821 | 2870 | 3620 | 6934 | 17245 |
| 124 | 39 | 8/6/2005 | 92.38 | 103.08 | 60359.4882 | 16978 | 3750 | 3620 | 29746 | 54094 |
| 125 | 54 | 8/16/2005 | 92.38 | 103.08 | 182052.5806 | 8695 | 9570 | 480 | 144410 | 163155 |
| 126 | 65 | 8/17/2005 | 92.38 | 103.08 | 66050.20048 | 7482 | 11110 | 360 | 40242 | 59194 |
| 127 | 48 | 8/20/2005 | 92.38 | 103.08 | 42634.5932 | 6257 | 14940 | 4220 | 12792 | 38209 |
| 128 | 79 | 9/15/2005 | 92.38 | 103.08 | 18182.38363 | 3785 | 4270 | 960 | 7280 | 16295 |
| 129 | 47 | 9/28/2005 | 92.38 | 103.08 | 230354.4538 | 19443 | 29100 | 3540 | 154360 | 206443 |
| 130 | 46 | 10/26/2005 | 92.38 | 103.08 | 135066.2663 | 26654 | 22430 | 20880 | 51082 | 121046 |
| 131 | 9 | 11/3/2005 | 92.38 | 103.08 | 57105.73977 | 3855 | 5900 | 9120 | 32303 | 51178 |
| 132 | 54 | 10/6/2018 | 102.42 | 103.08 | 329722.6462 | 62075.25 | 22560 | 24510 | 218466.3 | 327611.5 |
| 133 | 51 | 10/12/2018 | 102.42 | 103.08 | 303720.4092 | 74507.5 | 18875 | 17340 | 191053.3 | 301775.8 |
|  |  |  |  | Total | 16207686.4 |  |  |  |  |  |
|  |  |  |  | Average | 124674.5108 |  |  |  |  |  |

IPD, in-patient department
