# Supplementary material for: Burden of disease and productivity impact of Streptococcus suis infection in Thailand
Source: PLoS Negl Trop Dis. 2021 Jan 22;15(1):e0008985. doi: 10.1371/journal.pntd.0008985 (PMC7857555; doi:10.1371/journal.pntd.0008985)
Supplement: Attachment — Submitted filename: Cover letter to PLOS NTD Editor-in-Chief_2020-10-03.docx [file pntd.0008985.s002.docx]

Ajaree Rayanakorn, B.Pharm, MPH, Ph.D

Jeffrey Cheah School of Medicine and Health Sciences

Monash University Malaysia

Jalan Lagoon Selatan 47500

Bandar Sunway, Selangor Darul Ehsan

Malaysia

Date	3 October 2020

Elsio Wunder Jr, D.V.M., Ph.D.

Deputy Editor

*PLOS Neglected Tropical Diseases*

Dear Professor Elsio Wunder Jr,

On behalf of the authors, I am pleased to submit our revised manuscript entitled **"Burden of disease and productivity impact of *Streptococcus suis* infection in Thailand"** (PNTD-D-20-00652) for your consideration in *PLOS Neglected Tropical Diseases*.

We thank the reviewers for their insightful comments, which have led to an improved manuscript. The pages specified in this document relate to the revised manuscript with tracked changes. While the revised manuscript did not alter the overall conclusions of the study, it portrays a more robust and reliable estimate of the impact of *S.suis* infection on years of life lived, quality adjusted life years and productivity. Additionally, we have also added supporting information regarding *S.suis* acute treatment cost in supporting information (Table S1. A data set of 133 *Streptococcus suis* patients admitted with total IPD cost, page 21).

All authors having contribution met criteria for authorship. We attest that this work is original and has not been published and is not being considered for publication elsewhere. Please feel free to contact me with additional questions or concerns.

Point-by-point responses to all comments are as follows:

- **Methods**

**Reviewer #1: Minor revision: The introduction part is very short and most important limitations are missed that will support the claims by the authors. The motivation and gap of the study is not well defined. Regarding the method, the study design is properly meet the study objective. The target population is clearly defined.**

Response: Thank you for encouragement and for helpful feedback. We have revised the introduction section and we emphasized the rationale and gaps of the study. The revisions made are as follows.

**Revised version (lines 62-66, 75-84),**

**Lines 62-66**

Meningitis (68%), sepsis (25%) and infective endocarditis (12.4%) are major clinical manifestations of *S.suis* infection with a case fatality rate of 13% [1]. Long-term complications among survivors comprise sensorineural hearing loss (SNHL) with or without vestibular dysfunction and valvular heart disease. SNHL is the most common complication among *S.suis* meningitis patients which is typically bilateral and permanent [1, 2].

**Lines 75-84**

*S.suis* infection has been recognised as the cause of substantial loss in swine industry [6]. The only previous study on economic burden from *S.suis* infection in human was conducted in Vietnam [4]. In this study, the burden of disease was quantified in term of disability-adjusted life years (DALYs). The DALYs lost ranged 1,437-1,866 from 2011-2014 with the mean direct cost per episode of US$1,635 (95%CI 1,352-1,923), reflecting a significant economic impact [4]. However, long-term chronic consequences including cardiac complication after infective endocarditis (IE) and follow-up audiological testing were not captured in the model. The fact that the impact of *S.suis* infection is beyond acute infection involving long-term impairments including SNHL, valvular heart disease and premature mortality is of paramount importance to address the long-term sequelae of this sophisticated neglected infection.

**Reviewer #2: 1. Table 1: Please indicate why the different distributions were chosen for different parameters in the model? (How did results change when all varied parameter distributions were set to uniform)?**

Response: As per guidelines^1^ we used a gamma distribution for costs and beta distribution for utilities. The annual transition probabilities and productivity indices were assumed to be uniform distribution due to paucity of available data. In “Methods” section, we have revised manuscript accordingly as follows:

^1^ Briggs A, Sculpher M, Claxton K. Decision Modelling For Health Economic Evaluation 2006.

**Revised version (lines 201-204),**

Gamma and beta distributions were applied for costs and utilities respectively as per decision modelling guidelines [31]. The uniform distribution was assumed for annual transition probabilities and productivity indices.

**2. Probability of dying after IE: A probability cannot be > 1 (it is stated as 2.2 in the table). Can you clarify?**

Response: This is a relative risk (RR) which means those who suffered IE with long-term complications were 2.2 times more likely to die compared to the general population. We therefore have revised the wording as below:

**Revised version (lines 105-106)**

By contrast, those who suffered IE with long-term complications were 2.2 times more likely to die compared to the general population [7].

**3. IPD is not defined in the footnotes**

Response: Thank you for pointing this out. We have defined the full term in the footnotes accordingly as per suggestion by the reviewer, please see below:

**Revised version (lines 135-136)**

CT, computed tomography; IE, infective endocarditis; IPD, inpatient department; PALYs, productivity-adjusted life years; THB, Thai baht; VD, vestibular dysfunction

**4. Can the dollar values also be added to the cost parameters?**

Response: We have entered the dollar values in all the cost parameters (Table 1 Key input parameters) and revised the table as well as the footnotes. In addition, we have also amended Table 2 for all costs “Table 2 Base-case analyses results, with uncertainty intervals”. The revisions made are as follows.

**Revised version (lines 133-136, 212-215)**

**Lines 133-136**

**Table 1 Key input parameters**

| Parameters | Base-case value | Ranges | Distributions | Source |
| --- | --- | --- | --- | --- |
| Total population of Thailand in 2018 (million) | 68.416 | - | Fixed | Thailand Board of Investment. Thailand in Brief: Demographic.[13] |
| Annual disease incidence 2019 (per 100,000 population) | 0.457 | 0.420-0.493 | Fixed | Bureau of Epidemiology, Department of Disease Control, MoPH, Thailand*.[3] |
| Age of onset of acute infection (years) | 51.4 | 49.5-53.2 | Fixed | A systematic review and meta-analysis.[1] |
| Transitional probabilities |  |  |  |  |
| Case fatality rate | 0.128 | 0.090-0.180 | Uniform | A systematic review and meta-analysis.[1] |
| Infective endocarditis | 0.124 | 0.067-0.219 | Uniform | A systematic review and meta-analysis.[1] |
| Meningitis | 0.680 | 0.589-0.758 | Uniform | A systematic review and meta-analysis.[1] |
| Meningitis-hearing loss | 0.381 | 0.310-0.478 | Uniform | Retrospective cohort study [11]. |
| Markov |  |  |  |  |
| Probability of developing hearing loss | 0.680 | 0.544-0.816 | Uniform | Retrospective cohort study [11]. |
| Probability of developing hearing loss with VD | 0.120 | 0.096-0.144 | Uniform | Retrospective cohort study [11]. |
| Probability of developing complications from IE | 0.390 | 0.320-0.460 | Uniform | A systematic review and meta-analysis.[12] |
| Probability of dying after IE | 2.20 | 2.00-2.30 | Uniform | A nationwide cohort study.[7] |
| *Acute treatment cost*** |  |  |  |  |
| IPD per episode ($US) | 124,675 (4,132) | 11,337-40,4863 (376-13,418) | Gamma | Office of medical records and statistics, CMU Hospital.[Table S1] |
| *Chronic treatment cost* |  |  |  |  |
| IE after 1 month within 1^st^ year ($US) | 55,785 (1,849) | 44,628-66,942 (1,479-2,219) | Gamma | Thai Acute Coronary Syndrome (ACS) registry.[14] |
| IE following years ($US) | 15,934 (528) | 12,748-19,121 (422-634) | Gamma | Thai Acute Coronary Syndrome (ACS) registry.[14] |
| Hearing aids reimbursement ($US) | 12,769 (423) | 10,215-15,323 (339-508) | Gamma | National Health Security Office, Thailand.[15] |
| Audiometry ($US) | 220 (7.29) | 176-264 (6-9) | Gamma | Case series and Standard Cost List for Health Economic evaluation in Thailand.[8, 16] |
| CT Temporal bone ($US) | 7,344 (243) | 5,875-8,813 (195-292) | Gamma | Case series and Standard Cost List for Health Economic evaluation in Thailand.[8, 16] |
| *Health Utilities (Quality of Life Estimates)* |  |  |  |  |
| Hearing loss with or without VD | 0.58 | 0.34-0.81 | Beta | A multi-center, prospective study.[17] |
| Infective endocarditis (IE) | 0.67 | 0.40-0.94 | Beta | A single-center, prospective study.[18] |
| *PALYs* |  |  |  |  |
| Productivity index for no hearing loss | 0.96 | - |  | A national longitudinal study.[19] |
| Productivity index for hearing loss | 0.95 | 0.72-0.96 | Uniform | A national longitudinal study.[19] |
| Productivity index for hearing loss with VD | 0.92 | 0.72-0.96 | Uniform | A cross-sectional survey.[20] |
| Productivity index for IE | 0.94 | 0.45-0.96 | Uniform | A coronary heart disease model development study.[21] |

Costs are presented as Thai baht ($US), Quarter 3, year 2019 values	CT, computed tomography; IE, infective endocarditis; IPD, inpatient department; PALYs, productivity-adjusted life years; THB, Thai baht; VD, vestibular dysfunction

**Lines 212-215**

**Table 2 Base-case analyses results, with uncertainty intervals**

|  | Male | | Female | | Total | | Difference (%) | Lost per person |
| --- | --- | --- | --- | --- | --- | --- | --- | --- |
|  | **Infection** | **No infection** | **Infection** | **No infection** | **Infection** | **No infection** |  |  |
| YLLs | 5,482 | 6,376 | 1,680 | 1,954 | 7,162 | 8,329 | 1,167 (14.01) | 3.74 |
| Disc 3% | 3,707 | 4,296 | 1,136 | 1,316 | 4,844 | 5,612 | 769 (13.71) | 2.46 |
| Overall difference (uncertainty  ranges 95% CI) |  |  |  |  |  |  | 695 (12.38) to 841 (14.99) |  |
| QALYs | 3,422 | 4,352 | 1,049 | 1,334 | 4,471 | 5,686 | 1,214 (21.37) | 3.89 |
| Disc 3% | 2,323 | 2,955 | 712 | 906 | 3,035 | 3,861 | 826 (21.39) | 2.64 |
| Overall difference (uncertainty  ranges 95% CI) |  |  |  |  |  |  | 588 (15.23) to 1,098 (28.44) |  |
| PALYs | 5,219 | 6,139 | 1,600 | 1,881 | 6,818 | 8,020 | 1,202 (14.98) | 3.85 |
| Disc 3% | 3,529 | 4,136 | 1,082 | 1,267 | 4,611 | 5,404 | 793 (14.67) | 2.54 |
| Overall difference (uncertainty  ranges 95% CI) |  |  |  |  |  |  | 717 (13.27) to 867 (16.04) |  |
| Total treatment cost ($US million) | 35,813,161 (1.17) | 0 | 10,949,289 (0.36) | 0 | 46,762,449 (1.52) | 0 | -46,762,449  (-1.52) | -149,661 (-0.005) |
| Disc 3% ($US million) | 34,804,660 (1.13) | 0 | 10,639,082 (0.35) | 0 | 45,443,742 (1.48) | 0 | -45,443,742 (-1.48) | -145,441 (-0.005) |
| Overall difference (uncertainty  ranges 95% CI) |  |  |  |  |  |  | -55,222,281 to -38,966,206 (-$1,798,051 to -$1,268,749) |  |
| Broader economic cost in million ($US million) | 2,276 (74.10) | 2,677 (87.16) | 697 (22.71) | 820 (26.71) | 2,973.29 (96.81) | 3,497 (113.87) | 524 (17.06) | 1.68 (0.55) |
| Disc 3% ($US million) | 1,539 (50.11) | 1,804 (58.73) | 472 (15.36) | 553 (18) | 2,011 (65.47) | 2,356 (76.73) | 346 (11.26) | 1.11 (0.36) |
| Overall difference (uncertainty  ranges 95% CI) |  |  |  |  |  |  | 239,717,748 to 461,379,424 ($7,805,269 to $15,022,627) |  |
| Net cost ($US million) | 2,2340 (72.93) | 2,677 (87.16) | 687 (22.35) | 820 (26.71) | 2,927 (95.29) | 3,497 (113.87) | 570,760,094 (18.58) | 1.83 (0.59) |
| Disc 3% ($US million) | 1,504 (48.98) | 1,804 (58.73) | 461 (15.01) | 553 (18) | 1,965 (63.99) | 2,356 (76.73) | 391,277,289 (12.74) | 1.25 (0.41) |
| Overall difference (uncertainty  ranges 95% CI) |  |  |  |  |  |  | 281,879,015 to 509,098,159 ($9,178,050 to $16,576,361) |  |

Costs are presented as Thai baht ($US), Quarter 3, year 2019 values	YLLs, years of life lived; Disc, discount rate; QALYs, quality-adjusted life years; PALYs, productivity-adjusted life years, CI, confidence interval

**5. Is it reasonable to assume that the economic value of each PALY was equivalent to annual gross domestic product (GDP) per worker? Are there any data showing that persons exposed to and infected with *S. suis* might fall in a lower economic stratum?**

*Response: S.suis* is a zoonotic disease and there is a high prevalence among farmers and abattoir workers who might be in a lower socioeconomic status. The disease is occupational hazardous in western countries whereas cultural food habits involving raw pork consumption is an important risk factor in Asian countries including Thailand. According to a recent systematic review, major risk factors associated with *S.suis* infection include (1) exposure to pigs or pork, (2) pig-related occupation, (3) consumption of raw pork, and (4) male sex^1^. Underpinning a wide ranges of causes in acquiring *S.suis* infection apart from agricultural works. Disease mainly affects adults and elderly male of working age in Thailand but there is no information regarding occupation or income of *S.suis* cases reported according to the country Bureau of Epidemiology, Department of Disease Control. Therefore, using the economic value of each PALY to be equivalent to the annual gross domestic product (GDP) per full-time worker is justified*.*

^1^ Rayanakorn A, Goh B-H, Lee L-H, Khan TM, Saokaew S. Risk factors for Streptococcus suis infection: A systematic review and meta-analysis. Sci Rep. 2018;8(1):13358-. doi: 10.1038/s41598-018-31598-w. PubMed PMID: 30190575.

- **Results**

**Reviewer #1: The authors present the results based on the years of life lost, QALY and Productivity Adjusted Life Years (PALYs). But the analysis seems confusing. The authors present the analysis for example, years of life lost estimated in terms of both numbers of infection or non-infection and the number is considered as years of life lost. This is not clear.**

Response: Thank you for the comment. The numbers presented in the table is a summary for the whole population. In the results, we also described aggregated numbers for both males and females. The differences in the outputs of the two life tables (one each for the ‘*S.suis* infection cohort’ and the hypothetical ‘non *S.suis infection* cohort’) represented the years of life, QALYs and PALYs lost to *S.suis infection*. The following amendments were made to clarify results even further.

**Revised version (lines 216-229)**

# **Years of life lost to *S.suis* infection**

The differences in the outputs of the two life tables (one each for the ‘*S.suis* infection cohort’ and the hypothetical ‘non *S.suis infection* cohort’) represented the total years of life lost. The total discounted years of life lost to *S.suis* infection among the cohort was 769 (589 for males and 180 for females). At an individual level, this was equivalent to 2.46 years of life lost per person (2.46 for males and 2.47 females).

# **Quality-adjusted life years lost to *S.suis* infection**

The total discounted QALYs lost to *S.suis* infection among the cohort was 826 (632 for males and 194 for females). Overall, at individual level, this was equivalent to 2.64 QALYs lost (2.64 and 2.65 QALYs lost per male and female, respectively).

# **Productivity-adjusted life years lost to *S.suis* infection**

The total discounted PALYs lost to *S.suis* infection among the cohort was 793 (607 PALYs among males and 186 PALYs among females). Overall, 2.54 PALYs were estimated to be lost per person with *S.suis* infection (2.54 and 2.55 PALYs lost per male and female, respectively).

**Reviewer #2: 1. It is perhaps not surprising that there is a cost to the economy and a reduction in productivity in a scenario where there is *S. suis* infection versus a scenario where there is no *S. suis* infection. The bigger questions are:**

**a. How does the impact of *S suis* infection compare with the impact of other neglected tropical diseases in the country: E.g.: intestinal protozoan infections, leishmaniasis, zoonotic malaria (Plasmodium knowlesi infection), Melioidosis, rickettsial infections, and leptospirosis (and others?): https://journals.plos.org/plosntds/article?id=10.1371/journal.pntd.0003575#:~:text=Intestinal%20protozoan%20infections%20are%20widespread,selected%20rickettsial%20infections%2C%20and%20leptospirosis. “Neglected Tropical Diseases among the Association of Southeast Asian Nations (ASEAN): Overview and Update”**

Response: This is a very interesting point. We did attempt to compare the impact of *S.suis* infection with other neglected tropical diseases in the country. However, there is a paucity of the available national information concerning economic impact. The national information is mainly provided in the number of cases for each disease for the whole region without any granular information. We did look through all references listed in “Table 3. Summary of the major NTDs and malaria in the ASEAN countries” in the article and there was no country specific aggregated data.

Nevertheless, we did perform a search strategy to identify evidence about other neglected tropical diseases in the country with comparable information in terms of economic cost. We have created a new table and provided comparable information in the discussion section with regards to *S.suis* infection compared to other infectious diseases in Thailand.

**Revised version (lines 290-308)**

Table 3 shows the cost estimates from *S.suis* infection compared to other infections in Thailand. Dengue affects a large proportion of the population (N=81,000), equating to an annual loss of US$ 224 million [36]. In comparison, there are only 312 *S.suis* cases causing a loss of US$ 11.2 million annually (Table 3). However, the estimate loss per capita attributable to *S.suis* infection is far greater (US$ 36,033 per person) compared to dengue (US$ 5.03 per person) despite a nearly 260-fold difference in the number of annual cases.

When compared to pneumococcal disease, in terms of productivity impact, it is estimated that there are approximately 457,500 PALYs lost, which is equivalent to 0.15 PALYs lost per person, whereas there were 793 PALYs lost or 2.54 PALYs lost per person attributable to *S.suis* infection. The estimated high per-case impacts of *S.suis* reflects its long-term sequelae among infected people which warrants attention from policy makers of this disproportionate individual burden, as averting a single case would reduce substantial economic burden.

**Table 3 Cost estimates from *S.suis* infection compared to other infections in Thailand**

| Pathogen | Annual number of cases* | YLL loss | PALYs loss | | Broader economic cost ($2019)^a^ | |
| --- | --- | --- | --- | --- | --- | --- |
|  |  |  |  | **Per case** | **Annual cost in $2019 million** | **Loss per person ($2019)** |
| Dengue [34] | 81,000 | N.R. | N.R. | N.R. | 224 | 5.03 |
| Pneumococcal disease^b^[35] | N.R. | 453,401 | 457,498 | 0.15 | 5,586 | N.R. |
| *S.suis* | 312 | 769 | 793 | 2.54 | 11.26 | 36,033 |

Costs are presented as Thai baht ($US), Quarter 3, year 2019 values	YLLs, years of life lived; PALYs, productivity-adjusted life years, CI, confidence interval

*The numbers were derived from different years

^a^ Inflated to 2019 value

^b^ 2018 value

N.R., Not report

**b. What interventions are there to address *S. suis* infection and what is the cost-effectiveness of this intervention.**

**While I understand that the second question is beyond the scope of this paper, can you add a table summarizing the impact of the other NTDs? Is $11 million loss a big or small impact in comparison to other NTDs and health threats?**

**How much is currently invested in *S. suis* control?**

Response: Table 3 regarding cost estimates from *S.suis* infection compared to other infections in Thailand has been added. The amendments made have been summarized in the first question (a) above.

Regarding interventions to address *S.suis*, there was a food safety campaign implementation in Phayao province, northern Thailand in 2011 which resulted in a marked reduction of the disease incidence which we had already mentioned in “Discussion” section (lines 286-298 original version, lines 315-326 revised version) as below. The study did not present any information regarding the cost estimates of the interventions.

“Food safety campaigns or public health interventions to raise the disease awareness are potentially effective for disease control and prevention. According to a study on impact of a food safety campaign in Phayao province in northern, Thailand, the disease incidence was markedly reduced during the first two years after the program implementation but started to rise again in the third year [40]. This emphasizes the existence of deep-rooted cultural behaviour of raw pork consumption which is a major cause of infection and the need for continuous effective public health interventions to improve awareness among Thai population and healthcare policy makers. Raw pork eating practice was also identified as one of significant predictors (raw pork eating, meningitis, and vestibular dysfunction) of *S.suis* hearing loss which is a major sequelae among most surviving patients [41]. Considering significant economic burden incurred from *S.suis* infection which is far more than acute infection treatment, reducing incidence of new cases can potentially save costs substantially in a long term”.

- **Conclusions**

**Reviewer #1: The conclusions supported by the data and the authors discussed the limitations on their analysis clearly.**

Response: Thank you for encouragement and for helpful feedback.

**Reviewer #2: 1. Can the authors do a more convincing job convincing the reader that “*S.suis* infection imposes a significant economic burden both in terms of health and productivity”? Thailand has a GDP of over $540 Billion (https://tradingeconomics.com/thailand/gdp). The $11.3 million loss from the annual GDP is 0.002% loss of GDP which seems small. How does this compare to other NTDs and other health-related causes of GDP loss?**

Response: Thank you for the comment. We have now added more information to emphasize why the cost of this illness is significant which indicates the potential value of investment on effective preventive measures. Please see bellow amendments there were made in the manuscript page 14-15.

**Revised version (lines 290-301)**

“Table 3 shows the cost estimates from *S.suis* infection compared to other infections in Thailand. Dengue affects a large proportion of the population (N=81,000), equating to an annual loss of US$ 224 million [34]. In comparison, there are only 312 *S.suis* cases causing a loss of US$ 11.2 million annually (Table 3). However, the estimate loss per capita attributable to *S.suis* infection is far greater (US$ 36,033 per person) compared to dengue (US$ 5.03 per person) despite a nearly 260-fold difference in the number of annual cases.

When compared to pneumococcal disease, in terms of productivity impact, it is estimated that there are approximately 457,500 PALYs lost, which is equivalent to 0.15 PALYs lost per person, whereas there are 793 PALYs lost or 2.54 PALYs lost per person attributable to *S.suis* infection. The estimated high per-case impacts of *S.suis* reflects its long-term sequelae among infected people which warrants attention from policy makers of this disproportionate individual burden, as averting a single case would reduce substantial economic burden. “

**2. Is there any current investment in *S. suis* prevention? What is the annual cost of that prevention program currently or in the past?**

Response: As per response to the previous question (Results, Reviewer #2, b), there was a food safety campaign implementation in Phayao province, northern Thailand in 2011 which resulted a marked reduction of the disease incidence which we had mentioned in “Discussion” section (lines 315-326). However, no data is available regarding the cost of the interventions.

**3. Given that PALY is a new measure, can the authors indicate why it is important for this paper. If no other paper has reported a PALY, how can authors compare the PALY of *S. suis* with other NTDs or health threats? Do the authors think this should be a standard measure for future economic impact assessments? There is not much discussion of the value of PALY in the discussion section given its novelty.**

Response: Thank you for this valuable comment. PALYs is a relative new concept which has been introduced for only a few years to account on work productivity loss attributable to the disease at the population level. There are number of studies in Australia^1^, China^2^, Indonesia^3^, Bangladesh^4^ and Thailand^5^ that have applied PALY concept to quantify burden of disease in terms of productivity. PALYs are of same concept to QALYs, but instead of penalising years of life for time spent with reduced quality of life due to ill health, time spent with reduced work productivity is applied instead. This concept offers a more objective measure of associated impact on the broader economy, and we hope that it can be utilised as an additional metric to support decision making. We now have emphasized in the discussion section even further the novelty of the PALY metric.

^1^ Owen AJ, Maulida SB, Zomer E, Liew D. Productivity burden of smoking in Australia: a life table modelling study. Tobacco Control. 2019;28(3):297-304. doi: 10.1136/tobaccocontrol-2018-054263.

^2^ Hird TR, Zomer E, Owen A, Chen L, Ademi Z, Magliano DJ, et al. The impact of diabetes on productivity in China. Diabetologia. 2019;62(7):1195-203. Epub 2019/04/29. doi: 10.1007/s00125-019-4875-4. PubMed PMID: 31030220.

^3^ Uli RE, Satyana RPU, Zomer E, Magliano D, Liew D, Ademi Z. Health and productivity burden of coronary heart disease in the working Indonesian population using life-table modelling. BMJ Open. 2020;10(9):e039221. Epub 2020/09/12. doi: 10.1136/bmjopen-2020-039221. PubMed PMID: 32912956.

^4^ Afroz A, Hird TR, Zomer E, Owen A, Chen L, Ademi Z, et al. The impact of diabetes on the productivity and economy of Bangladesh. BMJ Glob Health. 2020;5(6). Epub 2020/06/14. doi: 10.1136/bmjgh-2020-002420. PubMed PMID: 32532757; PubMed Central PMCID: PMCPMC7295429.

^5^ Ounsirithupsakul T, Dilokthornsakul P, Kongpakwattana K, Ademi Z, Liew D, Chaiyakunapruk N. Estimating the Productivity Burden of Pediatric Pneumococcal Disease in Thailand. Applied Health Economics and Health Policy. 2020;18(4):579-87. doi: 10.1007/s40258-020-00553-0.

**Revised version (lines 273-289)**

“Our study is the first to quantify the impact of *S.suis* infection in terms of PALYs, which is a novel measure that accounts for productivity loss attributable to the disease. PALYs is a relatively new concept depicting the disease impact on work productivity which has been introduced for only a few years [9, 35, 36]. PALYs are of same concept to QALYs (widely used in health technology assessments), but instead of adjusting years of life for time spent with reduced quality of life due to ill health, time spent with reduced work productivity is applied instead. QALYs are limited in capturing wider effects on economic impact from the disease on work productivity [37] which is an important perspective on the impact of ill-health. Therefore, PALYs may offer a more objective measure in terms of of associated impact on the broader economy compared to QALYs, which may be seen as economic value loss to society.”

- **Summary and General Comments**

**Reviewer #1: The authors have addressed an important question on the burden of disease and productivity impact of *Streptococcus-suis* infection in Thailand. They estimated the health and economic burden of *S.suis* infection in terms of years of life lost, quality-adjusted life years (QALYs) lost, and productivity-adjusted life years (PALYs) lost and concluded that the infection have burden on health and productivity. After reading the manuscript I have the following minor comments.**

**The introduction part is very short and most important limitations are missed that will support the claims by the authors. The motivation and gap of the study is not well defined. Regarding the method, the study design is properly meet the study objective. The target population is clearly defined.**

**The authors present the results based on the years of life lost, QALY and Productivity Adjusted Life Years(PALYs). But the analysis seems confusing. The authors present the analysis for example for years of life lost estimated in terms of both numbers of infection or non-infection and the number is considered as years of life lost. This is not clear.**

**Figure captions are not included in the text of the manuscript.**

Response: Thank you for your comment. We have revised the introduction section and we emphasized the rationale and gaps of the study. The following revisions were made (lines 62-67, 75-84 in the revised manuscript) in the responses provided previously.

The results were described more clearly accordingly as per responses provided for “Results” section (lines 216-229 in the revised manuscript).

**Reviewer #2: As noted in the results section, It is perhaps not surprising that there is a cost to the economy and a reduction in productivity in a scenario where there is *S. suis* infection versus a scenario where there is no *S. suis* infection. The bigger questions are:**

**a. How does the impact of *S suis* infection compare with the impact of other neglected tropical diseases in the country: E.g.: intestinal protozoan infections, leishmaniasis, zoonotic malaria (Plasmodium knowlesi infection), Melioidosis, rickettsial infections, and leptospirosis (and others?): https://journals.plos.org/plosntds/article?id=10.1371/journal.pntd.0003575#:~:text=Intestinal%20protozoan%20infections%20are%20widespread,selected%20rickettsial%20infections%2C%20and%20leptospirosis. “Neglected Tropical Diseases among the Association of Southeast Asian Nations (ASEAN): Overview and Update”**

**b. What interventions are there to address *S. suis* infection and what is the cost-effectiveness of this intervention.**

**Is $11 million loss a big or small impact compared with other NTDs and health threats? The paper needs additional tables that allow comparison with other NTDs and health threats to place the findings of the paper in context.**

Response: Thank you for this insightful comment. We have revised our manuscript to address your comments accordingly. Please refer to the details in our responses to your comment in “Results” and “Conclusions” sections indicated above.

Yours sincerely,

Ajaree Rayanakorn and behalf of all co-authors

Ajaree Rayanakorn, B.Pharm, MPH, Ph.D

Jeffrey Cheah School of Medicine and Health Sciences

Monash University Malaysia

Email: ajaree.rayanakorn@monash.edu
